# Heat vs. Fatigue: Hyperthermia as a Possible Treatment Option for Myalgic Encephalomyelitis/Chronic Fatigue Syndrome (ME/CFS)

**DOI:** 10.3390/ijms26115339

**Published:** 2025-06-01

**Authors:** Barbara Hochecker, Katja Matt, Melanie Scherer, Alica Meßmer, Alexander von Ardenne, Jörg Bergemann

**Affiliations:** 1 Department of Life Sciences, Albstadt-Sigmaringen University of Applied Sciences, Anton-Günther-Strasse 51, 72488 Sigmaringen, Germany; matt@hs-albsig.de (K.M.); schererm@hs-albsig.de (M.S.); bergemann@hs-albsig.de (J.B.); 2 Von Ardenne Institute of Applied Medical Research GmbH, 01324 Dresden, Germany; ceo@ardenne.de

**Keywords:** hyperthermia, myalgic encephalomyelitis/chronic fatigue syndrome (ME/CFS), human PBMCs (peripheral blood mononuclear cells), autophagy, mitochondrial function, mRNA expression

## Abstract

The aetiology and pathophysiology of myalgic encephalomyelitis/chronic fatigue syndrome (ME/CFS) have not yet been clarified. Its exact diagnosis is also difficult because it has no biomarkers. This lack of knowledge leads to difficulties in treating the disease. In our work, we are attempting to counteract this problem by analysing the central cellular mechanisms in ME/CFS patients and comparing them with those of healthy individuals. This pilot study provides a small glimpse into the journey of nine people with ME/CFS—more specifically, how their peripheral blood mononuclear cells (PBMCs) responded immediately after a session of whole-body hyperthermia (WBH). The clinical effect of WBH has already been investigated in other studies on the treatment of ME/CFS, and these studies have provided valuable insights into its potential benefits. The present study is concerned with the investigation of cellular parameters, namely autophagy, mitochondrial function and mRNA expression, before and after WBH. The results suggest that ME/CFS patients may have higher autophagy-related protein light chain 3 (LC3)-II levels and increased mitochondrial function compared with healthy individuals. A whole-body hyperthermia session could lead to a reduction in LC3-II levels, resulting in a reversion to the levels observed in healthy donors. In the case of mitochondrial parameters, hyperthermia could lead to an increase in the measured parameters. This pilot study is a continuation of a previously published study in which only the isolated cells of ME/CFS patients and a healthy control group were treated with hyperthermia.

## 1. Introduction

One main symptom of myalgic encephalomyelitis/chronic fatigue syndrome (ME/CFS) is fatigue. This fatigue is often profound and not the result of prolonged, unusual or excessive exertion, and it is not significantly relieved by rest. The fatigue is accompanied by cognitive impairment, orthostatic intolerance and immunological symptoms such as a persistent sore throat, painful and enlarged lymph nodes and fever. Another key feature of this multisystemic disorder is post-exertional malaise (PEM), which is a worsening of symptoms after trivial physical or cognitive exertion, often with a delayed onset and an abnormally delayed recovery. ME/CFS can severely affect patients’ ability to live normal lives [1,2]. Unfortunately, this serious disease is not yet sufficiently understood. There are many gaps in knowledge regarding its aetiology and pathophysiology. Furthermore, there are no biomarkers, and all diagnostic criteria are based on clinical symptoms and the exclusion of other fatigue disorders [3].

Without truly understanding this disease, no causal therapy is possible. Instead, there are either treatment options to alleviate the symptoms or methods to prevent the symptoms from worsening. Pharmaceutical therapies to alleviate the symptoms include painkillers, anti-inflammatory drugs and medication for sleep disturbances [4]. One method of prevention for PEM is Adaptive Pacing Therapy (APT). Patients receiving APT are instructed to set reasonable goals for their daily activities to avoid possible overexertion by establishing a balance between activity and rest. APT patients who are functioning within their individual limits then gradually increase their daily activity levels [5]. The overall aim is to increase performance levels, to raise the threshold before exhaustion sets in or even to take a step back to ‘normal/healthy’ energy levels.

Our approach in this study is a cellular investigation of the influence of a form of therapy that has been used successfully for many years, mainly in cancer therapy [6,7,8,9,10,11]: heat treatment (or hyperthermia). Interestingly studies on whole-body hyperthermia (WBH) and ME/CFS patients show that perceived fatigue decreased significantly after therapy. In those hyperthermia studies, Waon therapy was used, which consisted of a 15-min sauna treatment at 60 °C. Other results were that negative mood, including anxiety, depression and fatigue, as well as performance status improved significantly after the therapy. In addition, these Japanese studies on ME/CFS-patients concluded that the hyperthermia treatment increased cerebral blood flow (CBF) [12,13]. This improvement in blood flow is of particular interest, as a reduction in blood flow has already been observed in ME/CFS patients in several studies. As early as 1995, Costa et al. showed reduced blood flow in the brain stem in ME/CFS patients compared with healthy donors [14]. A study of 400 people with ME/CFS revealed that their cerebral blood flow decreases by more than three times when they are upright compared with the supine position. This decrease in blood flow is associated with symptoms of orthostatic intolerance [15]. Another finding shown in 2011 by Newton et al. for ME/CFS is the impaired dilation of blood vessels or endothelial dysfunction [16].

The cellular and molecular mechanisms underlying the benefits of hyperthermia are poorly understood. For this reason, in a previous study, we compared key parameters of cell function in cells from healthy donors and cells from ME/CFS patients after hyperthermia. In this previous study, donor peripheral blood mononuclear cells (PBMCs) were isolated after blood collection and then treated ex vivo with a special heating device known as the ‘IRAcubator’. Our results showed an influence of hyperthermia on autophagy, mitochondrial function and the expression of various mRNAs [17]. Based on the promising results of our first study, we wanted to further investigate the immediate effect of a session of whole-body hyperthermia on autophagy and mitochondrial function in ME/CFS patients, which is described in this pilot study.

Autophagy is a lysosomal process that enables the degradation of cytoplasmic components and organelles and can be divided into six phases: initiation, nucleation, elongation, maturation, fusion and degradation. Autophagy occurs either as a mechanism to remove potentially harmful substances, such as damaged organelles, or as a source of metabolic products that are essential for maintaining cell metabolism [18]. Dysregulated autophagy has been associated with several diseases and pathophysiological conditions, including autoimmune, cardiovascular, neurodegenerative, metabolic, rheumatic and pulmonary diseases, as well as ageing [19]. To measure the autophagy marker light chain 3 (LC3)-II, a cell-based assay was used in which membrane-bound LC3-II was measured with an FITC-conjugated antibody and subsequent flow cytometry.

In addition, the mitochondrial parameters of basal respiration, adenosine triphosphate (ATP) production, maximal respiration and free respiratory capacity were determined by measuring the oxygen consumption rate of PBMCs. These mitochondrial parameters are of interest because the main function of mitochondria is to generate energy in the form of adenosine triphosphate through oxidative phosphorylation. Consequently, the measurement of mitochondrial function is crucial when it comes to fatigue.

The second system for investigating autophagy and mitochondrial function is the quantification of the mRNA of genes coding for the proteins involved in these processes. In the case of autophagy, different stages of the autophagy process were quantified, namely ULK1 (initiation), Beclin 1 (nucleation) and ATG7 and MAP1LC3B (elongation). The protein kinase AMP-activated catalytic subunit alpha 1 (AMPK1α), the forkhead box O3 (FOXO3) and the protein sirtuin 1 (SIRT1) are significantly involved in the regulation of the autophagy process and so were also analysed [20,21,22]. To investigate the function of mitochondria, the mRNA of several mitochondria-associated genes was analysed. One of them is the gene encoding sirtuin 3 (SIRT3), which is localised in the mitochondria and which reduces the production of reactive oxygen species (ROS), stabilises mitochondrial structure and function and supports the stress response [23]. Mitochondrial transcription factor A (TFAM) is another gene that codes for a protein located in the mitochondria and plays a key role in energy metabolism [24]. The NADH-ubiquinone oxidoreductase 75 kDa subunit S1 (NDUFS1), which forms the largest part of complex I [25], and the powerful antioxidant superoxide dismutase 2 (SOD2) [26] were also investigated. In addition, the central modulator of the immune system, interleukin-10 (IL-10), and the gene of the heat shock protein family A (Hsp70), member 5 (HSPA5), were measured.

The aim of this paper is to evaluate the described cellular parameters immediately after a WBH session in comparison with the data from the first publication and to determine a possible explanation for the data obtained in the form of a hypothesis.

## 2. Results

The purpose of this pilot study was to investigate the influence of a single session of whole-body hyperthermia (one-hour WBH therapy with T_c max_ = 39 °C) on the central cellular mechanisms in the peripheral blood mononuclear cells (PBMCs) of nine ME/CFS patients. In addition, data from healthy donors from previous studies were visualised to compare basal levels in healthy donors and ME/CFS patients. The results of the measurements of autophagy, mitochondrial respiration and mRNA expression are presented and described in the following sections. To enable better comparison and evaluation of all results achieved, each ME/CFS patient has been assigned a unique symbol, which can be found in Table 1.

### 2.1. Autophagy

**Figure 1:**
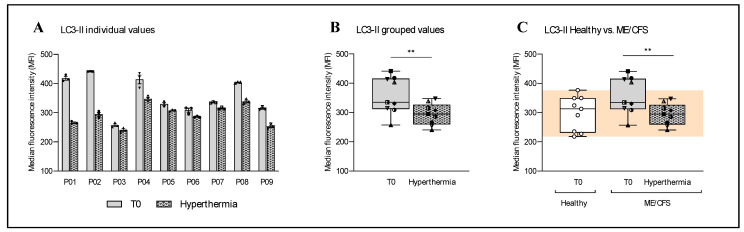
Cell-based investigation of the immediate influence of hyperthermia (one-hour WBH therapy with T_c max_ = 39 °C) on autophagy in the PBMCs of nine ME/CFS patients by measuring LC3-II levels. (**A**) Individual analysis of the nine ME/CFS patients showed a decrease in LC3-II levels after a session of whole-body hyperthermia in all participants (descriptive, without statistical tests). (**B**) The grouped LC3-II values of all nine participants showed a 17.84% decrease in the mean value, which was statistically significant (** *p* = 0.0065; paired *t*-test), as a result of the treatment. (**C**) A comparison of ME/CFS patients’ data before and after treatment with the basal levels in healthy donors (n = 9) showed an elevated (by 20.01%) basal level of LC3-II in ME/CFS patients with a *p*-value of 0.0538 (unpaired *t*-test) (Table S1). The orange area shows the range of values for the healthy comparison group.

The cell-based assessment of autophagy was performed by quantifying the autophagy marker light chain 3 II (LC3-II) and is shown in Figure 1 below. Each of the nine participating ME/CFS patients showed a reduction in their LC3-II levels after one session of WBH at a maximum core body temperature of 39 °C (Figure 1A; descriptive, without statistical tests). The grouped values of these nine ME/CFS participants confirm this reduction, with a mean percentage reduction of 17.84% and a statistical significance of ** *p* = 0.0065, as measured with a paired *t*-test (Figure 1B). The comparison of basal data from nine ME/CFS patients and nine untreated healthy donors is shown in part C of Figure 1. The data from the measured participants showed elevated LC3-II levels in ME/CFS patients compared with healthy donors, with a mean percentage increase of 20.02% and a *p*-value of 0.0538, as measured by an unpaired *t*-test.

### 2.2. Mitochondrial Function

**Figure 2:**
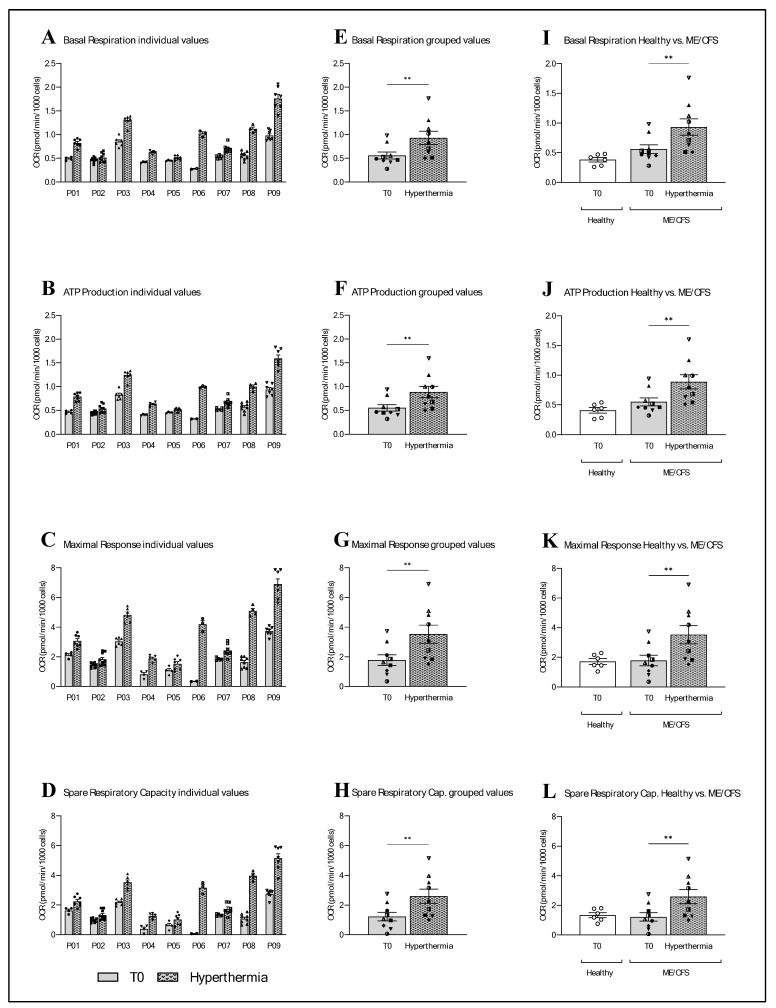
All the measured mitochondrial parameters increased in the PBMCs of the nine ME/CFS patients immediately after whole-body hyperthermia. (**A**–**D**) All nine participants showed an increase in the parameters of basal respiration, ATP production, maximal response and spare respiratory capacity (descriptive, without statistical tests). (**E**–**H**) The basal respiration of the grouped data of the nine ME/CFS patients increased by 66.60% with WBH (** *p* = 0.0040; paired *t*-test). ATP production increased by 61.41% (** *p* = 0.0023; paired *t*-test), the maximal response by 97.88% (** *p* = 0.0071; paired *t*-test) and the spare respiratory capacity by 112.35% (** *p* = 0.0086; paired *t*-test). (**I**–**L**) Comparison of the grouped T0 data of ME/CFS patients (n = 9) with an untreated healthy control group (n = 6) showed an elevation of 46.43% in basal respiration in ME/CFS patients compared with the control group (*p* = 0.0869; unpaired *t*-test). ATP production was 35.25% higher (*p* = 0.145; unpaired *t*-test) and the maximal response was 3.52% higher (*p* = 0.8992; unpaired *t*-test) in ME/CFS patients. The spare respiratory capacity was 8.71% lower (*p* = 0.7642; unpaired *t*-test) in ME/CFS patients (Table S2).

All the mitochondrial parameters of the nine ME/CFS patients, namely basal respiration, ATP production, maximal respiration and spare respiratory capacity, were affected by a session of whole-body hyperthermia (Figure 2). More specifically, all nine ME/CFS patients showed an increase in all the mitochondrial parameters examined. This is evident in both the individual data and the grouped data. In the grouped data, basal respiration was shown to have increased by 66.60% (** *p* = 0.0040; paired *t*-test), ATP production by 61.41% (** *p* = 0.0023, paired *t*-test), the maximal response by 97.88% (** *p* = 0.0071; paired *t*-test) and the spare respiratory capacity by 112.35% (** *p* = 0.0086; paired *t*-test). A comparison of the grouped data from the nine ME/CFS patients at T0 with data from the six healthy donors showed that basal respiration and ATP production was elevated by 46.43% (*p* = 0.0869; unpaired *t*-test) and 35.25% (*p* = 0.145; unpaired *t*-test), respectively, in ME/CFS patients. The T0 values of the maximal response were also 3.52% higher (*p* = 0.8992; unpaired *t*-test) in ME/CFS patients, whereas the spare respiratory capacity was 8.71% lower (*p* = 0.7642; unpaired *t*-test) in ME/CFS patients compared with the healthy control group.

### 2.3. mRNA Expression

**Figure 3:**
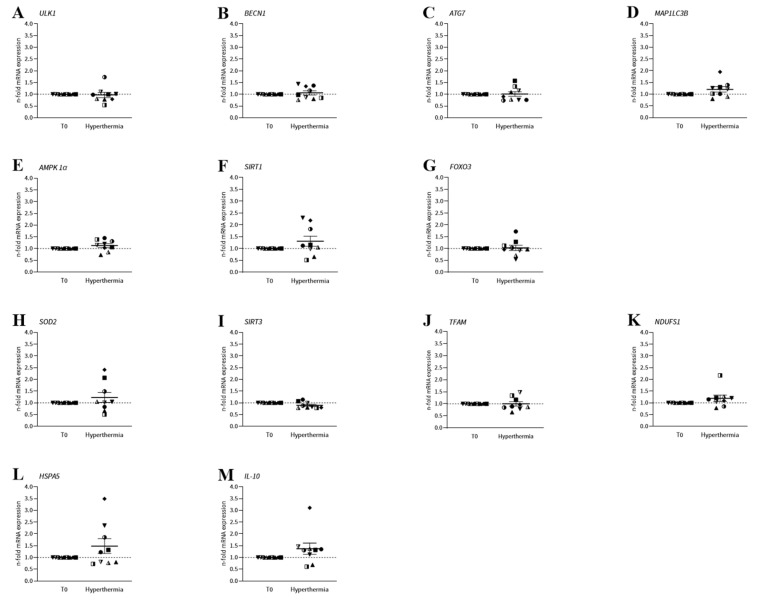
Immediate influence of a session of WBH on mRNA expression in PBMCs from nine ME/CFS patients. (**A**–**D**) Among the genes regulating autophagy, WBH increased *BECN1* by 6.11% (*p* = 0.5147; paired *t*-test), *ATG7* by 1.00% (*p* = 0.9221; paired *t*-test) and *MAP1LC3B* by 20.33% (*p* = 0.1125; paired *t*-test). The only decrease was in *ULK1*, which decreased by 3.00% (*p* = 0.7925; paired *t*-test). (**E**–**G**) The autophagy-related genes *AMPK1α*, *SIRT1* and *FOXO3* increased by 13.00% (*p* = 0.1429; paired *t*-test), 30.89% (*p* = 0.1886; paired *t*-test) and 2.78% (*p* = 0.8110; paired *t*-test), respectively, with hyperthermia. (**H**–**K**) The mitochondrial genes *SOD2*, *NDUFS1* and *TFAM* increased by 22.89% (*p* = 0.3162; paired *t*-test), 19.67% (*p* = 0.1602; Wilcoxon test) and 0.11% (*p* = 0.9905; paired *t*-test), respectively. *SIRT3* mRNA expression decreased by 10.22% (*p* = 0.0508; Wilcoxon test). (**L**,**M**) *HSPA5* and *IL-10* increased by 48.33% (*p* = 0.2188; Wilcoxon test) and 37.33% (*p* = 0.1367; Wilcoxon test), respectively, as a result of hyperthermia (Table S3).

In addition to the cellular assays of autophagy and mitochondrial function, mRNA expression was monitored to observe any alterations in the molecular mechanisms of PBMCs induced by whole-body hyperthermia (Figure 3). In this pilot study, we focused on the genes of proteins that play a key role in the regulation of the autophagy process or that are directly involved in the mechanisms of autophagy. We also analysed mitochondrial genes as well as a gene that encodes a heat-shock protein that should react to heat exposure and a gene that plays an essential role in the immune system. For the genes *ULK1*, *BECN1*, *ATG7* and *MAP1LC3B*, which are directly involved in autophagy, *ULK1* decreased by 3.00% as a result of hyperthermia (*p* = 0.7925; paired *t*-test), *BECN1* increased by 6.11% (*p* = 0.5147; paired *t*-test), *ATG7* by 1.00% (*p* = 0.9221; paired *t*-test) and *MAP1LC3B* by 20.33% (*p* = 0.1125; paired *t*-test). The autophagy-related genes *AMPK1α* and *SIRT1* increased by 13.00% (*p* = 0.1429; paired *t*-test) and 30.89% (*p* = 0.1886; paired *t*-test), respectively, after one session of WBH. *FOXO3* also increased by 2.78% (*p* = 0.8110; paired *t*-test). The mitochondrial genes *SOD2*, *NDUFS1* and *TFAM* increased by 22.89% (*p* = 0.3162; paired *t*-test), 19.67% (*p* = 0.1602; Wilcoxon test) and 0.11% (*p* = 0.9905; paired *t*-test), respectively. *SIRT3* mRNA expression decreased by 10.22% (*p* = 0.0508; Wilcoxon test). The two additional genes, *HSPA5* and *IL-10*, increased by 48.33% (*p* = 0.2188; Wilcoxon test) and 37.33% (*p* = 0.1367; Wilcoxon test), respectively, as a result of hyperthermia.

## 3. Discussion

The goal of our research is to gain more knowledge about the severe disease ME/CFS to find a way to help those people who suffer from it. To this end, we are investigating key cellular mechanisms in ME/CFS patients, namely autophagy and mitochondrial function as well as their underlying molecular mechanisms by measuring the mRNA expression of the genes associated with these mechanisms. In addition, we are investigating the influence of the possible treatment method of hyperthermia on these processes. In a previous study, we analysed these mechanisms in isolated peripheral blood mononuclear cells (PBMCs) from healthy donors and ME/CFS patients with and without hyperthermia of the isolated cells. Given the promising outcome of this initial study, we wondered how whole-body hyperthermia treatment, using the IRATHERM^®^1000, of ME/CFS patients, rather than just their PBMCs, would affect the cellular mechanisms under investigation. The results of this investigation are provided in the context of the present pilot study. Surprisingly, the results of whole-body hyperthermia differ greatly from the results of the first study, in which only the cells were irradiated. These results, among others, are discussed below.

Although the results of wIRA-irradiated PBMCs and whole-body hyperthermia are different, measurements of the basal levels of autophagy-related protein light chain 3 (LC3-II) and mitochondrial respiration before treatment show the same pattern in the first and second study. The results of the first study showed significantly elevated LC3-II values and a slight non-significantly higher mitochondrial function in ME/CFS patients compared with a healthy control group. Those results are confirmed by the present pilot study, as the LC3-II levels in ME/CFS patients were 20.01% higher than those in healthy donors. In terms of mitochondrial function, the oxygen consumption rate in the mitochondrial parameters of basal respiration and ATP production was elevated by 46.43% and 35.25%, respectively, in ME/CFS patients. The parameters of maximal response and spare respiratory capacity showed no noteworthy differences between the two groups (Figure 2). As the name suggests, basal respiration ensures oxygen consumption at the basic level. ATP production refers to the consumption of oxygen for the regeneration of ATP. From these results, we can conclude that the mitochondria of ME/CFS patients consume more oxygen at a basal level and for ATP production compared with healthy donors. Either the mitochondria of ME/CFS patients are more active than the mitochondria of healthy donors or they have a dysfunction in their process that means that they need more oxygen to produce the same amount of ATP. However, the maximal response, which measures oxygen consumption under stress, and the spare respiration capacity, which indicates the ability to respond to an energy demand and is an indicator of cell fitness or flexibility, showed no difference between ME/CFS patients and the control group. This lack of difference does not indicate mitochondrial dysfunction. Lawson et al. also compared blood cells from ME/CFS patients with those from a healthy control group and found that ATP levels were higher in the patients, and the enzymatic activities of the complexes in the electron transport chain remained intact [20]. Another study from 2019, which investigated the activity of complexes I, II and IV in skeletal muscle and PBMCs, also confirmed these results, finding that there were no significant differences in the activity of the individual mitochondrial complexes [21]. Both these studies, and our study, initially assumed mitochondrial dysfunction, and in all cases, the mitochondrial activity proved us wrong. So, all these studies came to the same conclusion: mitochondrial function does not appear to be the problem. This could either be related to a pathological mechanism in which more ATP is produced from non-mitochondrial sources, or the cause is upstream of the mitochondrial respiratory chain [20,21].

However, it is not only mitochondrial function that behaves differently than expected. The investigation of autophagy by quantifying LC3-II also showed an unexpected result. As in our first study, the results of this pilot study show significantly higher LC3-II basal levels in ME/CFS patients than in the healthy control group (Figure 1). These results contradict our original hypothesis of impaired autophagy as well as the results of another study demonstrating dysfunction of the autophagic process. However, it is worth noting that we only found one other study on autophagy in ME/CFS. The findings of that study indicate that the autophagy-related protein ATG13 is present at elevated levels in the serum of ME/CFS patients. This suggests that there is an impairment in the metabolic processes of autophagy [22]. Impaired autophagy could explain the results of another study showing increased protein aggregation in ME/CFS patients [23]. However, the increased amount of protein aggregation raises the following question: what came first? Is the increased protein aggregation a result of impaired autophagy, or is autophagy more active in ME/CFS to counteract this phenomenon?

The fact that basal levels were similar in ME/CFS patients and healthy donors in both studies gives us a starting point for discussing the effect of wIRA treatment. Interestingly, these results show no similarity at all, but on the contrary, the exact opposite response is seen. While wIRA treatment of isolated PBMCs significantly increased LC3-II levels, whole-body hyperthermia in ME/CFS patients led to a significant decrease in LC3-II levels. Furthermore, while the mitochondrial function parameters decreased with wIRA treatment of PBMCs, a whole-body hyperthermia session led to a significant increase in the same parameters. So, there is a big difference in the way the same cells respond when only the PBMCs are treated or when they are surrounded by a whole organic system during treatment. As mentioned before, we assume that the function of the mitochondria is not disturbed, but that there is a problem with the upstream sources for the mitochondria. A crucial source required for oxidative phosphorylation is oxygen. Without oxygen, cellular respiration—also known as aerobic respiration—cannot function smoothly [24]. We further assume that without sufficient oxygen, the transfer of electrons to oxygen is not possible, and electrons accumulate in the mitochondrial transport chain. Oxygen is transported to the cells via our bloodstream through the erythrocytes. Interestingly, this finding of a link between reduced blood flow in ME/CFS patients is not new. A 2012 study by Newton et al. showed impaired dilation of blood vessels in ME/CFS patients [16]. A correlation between the severity of endothelial dysfunction and the severity of ME/CFS disease was demonstrated by Scherbakov et al. [25]. It is hypothesised that impaired blood circulation reduces tissue oxygenation in ME/CFS patients. The lack of oxygen in ME/CFS patients could explain their higher baseline level of mitochondrial function compared with that of healthy individuals, as the cells are resupplied with sufficient oxygen during the assay conduction, and all accumulated electrons in the mitochondrial transport chain are utilised for ATP production in a short time. As already mentioned, whole-body hyperthermia is associated with increased oxygen partial pressure and improved blood circulation [12,26]. This means that whole-body hyperthermia restores the oxygen supply and reactivates mitochondrial function. The higher LC3-II levels in ME/CFS patients compared with healthy donors could also be explained by the hypoxia theory. Autophagy is activated as a survival mechanism under hypoxia by the hypoxia-inducible factor (HIF), as has been shown by Bellot et al. in normal and cancer cell lines [27]. After whole-body hyperthermia, oxygen supply is restored and LC3-II levels decrease to the healthy donor range. When only the PBMCs are treated with wIRA irradiation, the oxygen factor plays no role, leading to an even stronger activation of autophagy due to heat stress, as shown in the work of McCormick et al. [28].

To obtain a profile of the molecular mechanisms of autophagy and mitochondrial function, the mRNA expression of the associated genes was also analysed. The genes directly involved in autophagy, *ULK1*, *BECN1*, *ATG7* and *MAP1LC3B*, as well as the genes *AMPK*, *SIRT1* and *FOXO3*, which are involved in the regulation of autophagy, were observed in association with autophagy. In this pilot study, our results showed a decrease in LC3-II, leading to the hypothesis that the gene expression of genes involved in autophagy also decreases after whole-body hyperthermia. Looking at the mRNA expression of *ULK1*, *BECN1* and *ATG7*, this cannot be confirmed, as they remained at the same level during treatment. *MAP1LC3B* showed an increase of 20.33% in mRNA after whole-body hyperthermia. Amirkavei et al. observed a time-dependent response after heat shock (42 °C) in the regulation of central-autophagy-associated genes, which may explain our mRNA expression profile. In the case of *BECN1*, an increase in mRNA was only observed after 12 h of recovery and even occurred after 24 h, which is far outside our time frame. Neither was any change in *ATG7* mRNA expression observed at any of the three time points. *MAP1LC3B* was declared an ‘early-response gene’, because the strongest response occurred 3 h after heat shock, which is also consistent with our data [29]. There were increases in the regulating genes *AMPK1α* and *SIRT1* of 13.00% and 30.89%, respectively, while *FOXO3* remained at the same level. Overall, none of the genes associated with autophagy were significantly affected by whole-body hyperthermia. This led to the conclusion that autophagy is mainly controlled at the translational level at the time point investigated. The mRNA of the genes *SOD2*, *SIRT3*, *TFAM* and *NDUFS1* was measured in connection with mitochondrial function. Due to the increased mitochondrial function caused by whole-body hyperthermia, an increase in mitochondrial genes was expected. In the case of *SOD2* and *NDUFS1*, increases of 22.89% and by 19.67%, respectively, were observed, confirming our hypothesis. Since increased mitochondrial function increases ROS, it is not surprising that the mRNA expression of the potent antioxidant *SOD2* also increased by 22.89%. The measured data of the n-fold mRNA expression of *SOD2* also show a high degree of scatter. This indicates a different response of the donors to heat stress. A comparison of these data in a study on mammalian cells (buffalo granulosa cells) at different temperatures showed a slight decrease in *SOD2* mRNA expression at 39.5 °C, while at 40.5 °C, there was a strong increase in *SOD2* expression [30]. This shows the fine regulation of this heat response and explains the variability in the donors. *SIRT3* and *TFAM* showed no change in mRNA expression after whole-body hyperthermia. In addition, the mRNA of *HSPA5*, the gene of the HSP70 protein, and *IL-10* were measured, and both showed increases of 48.33% and 37.33%, respectively, due to whole-body hyperthermia. Amirkavei et al. also showed heat-activated expression of *HSP70* [29]. The increased *IL-10* expression could reflect the immunomodulatory effect of whole-body hyperthermia and was also evident in other studies [31,32].

To better evaluate the results presented, it is important to recognise the limitations of this pilot study. The first limitation is that we cannot guarantee that all ME/CFS patients were subjected to exactly the same treatment conditions. The planned treatment condition was one-hour WBH therapy with T_c max_ = 39 °C, but this treatment involved significant stress to the body, especially for ME/CFS patients. Therefore, it is possible that not all patients were treated at these exact parameters, and it was, at times, necessary to lower the temperature a little or to abort the session before the full hour was reached. However, it is important to bear in mind that this had no negative influence on the effect of the therapy, as a change was measured in at least one of the parameters analysed in all nine participants. Furthermore, this has nothing to do with the therapy itself but is dependent on the physical reaction of the patient. This influencing factor cannot be controlled unless the patient is forced to endure the temperature regardless of how he or she is feeling. That would not be ethically justifiable under any circumstances. Furthermore, this was a small study, which minimises the statistical significance. However, as already mentioned, an effect could be seen in all nine participants, which is exceptional and speaks strongly in favour of the treatment. We consider it a strength of this study. The small number of subjects is due to our requirement to analyse the samples as quickly as possible (within a few hours) as we were performing cellular assays, and we cannot exclude the possibility of the cells being affected by hours of transport, which is also a strength of this study. However, we are geographically limited, which minimised the number of participants. This pilot study also only investigated the immediate cellular response to a WBH session. Further studies that also observe the long-term effects, disease progression and the effects of multiple WBH sessions are needed. We hope that this small study will inspire further studies with a larger group of participants (cases and controls), as validating the obtained results in an extended cohort is an absolute requirement. In addition, the underlying mechanisms of the induced changes in autophagy and mitochondrial function are still unclear, so further experiments are needed to investigate these. A further limitation is that all the participating ME/CFS patients were undergoing medical treatment, and the influence of their individual treatments could not be determined. However, it would also be ethically unacceptable to deny patients their treatment to participate in this study. Another weakness is that there is little clinical data on the effect of WBH on ME/CFS patients, and we failed to complete standardised questionnaires. Nevertheless, some of the patients who participated in this study reported an improvement in certain symptoms as a result of the therapy. These include an improvement in flu-like symptoms, such as fewer swollen lymph nodes, a reduction in recovery time after an effort, and an overall greater ability to cope with stress as well as fewer restrictions in daily life.

Furthermore, it is difficult to capture the dynamic process of autophagy using static measurement methods. The method used measures cellular autophagosomes by labelling the marker LC3B-II, which generally indicates the extent of cellular autophagy activity. However, the accumulation of autophagosomes is not always indicative of autophagy induction. It may also indicate blockage of autophagosomal maturation and termination of the autophagy pathway by lysosomal degradation. However, in each test, we performed an additional control with chloroquine, which prevents lysosomal degradation, and achieved an increase in LC3B-II levels; thus, a malfunction in lysosomal degradation can be excluded. It is also recommended to use more than one method to measure autophagy. Therefore, we decided to measure the mRNA expression of autophagy-related genes, as described above [33].

**Figure 4:**
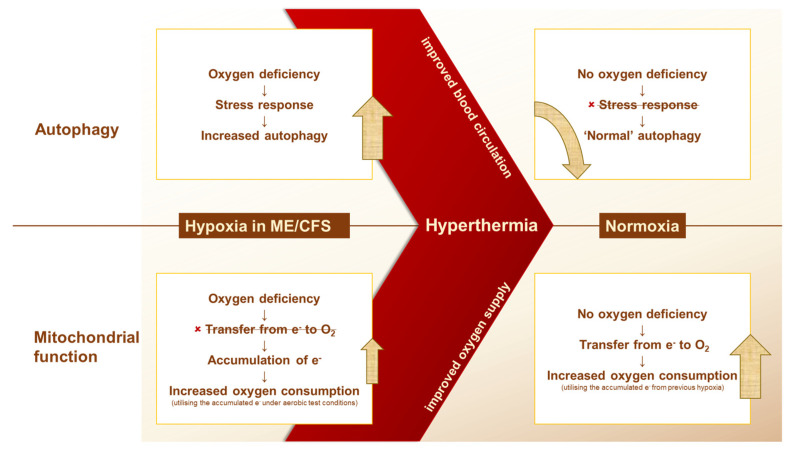
Schematic overview of our hypothesis. The disturbed blood flow in ME/CFS leads to hypoxia and stress-induced activation of autophagy as well as to an accumulation of electrons and, thus, to higher oxygen consumption under aerobic experimental conditions. Whole-body hyperthermia restores normoxia, leading to a decrease in autophagy due to the termination of the stress response and to even higher oxygen consumption due to the utilisation of the accumulated electrons under aerobic conditions.

To summarise, the results of our research lead us to the hypothesis shown in Figure 4. It implies the idea of a disturbed blood circulation in ME/CFS patients, which leads to hypoxia in the cells. This hypoxia leads to a stress-induced activation of autophagy. In the case of mitochondrial function, this leads to an accumulation of electrons in the respiratory chain, as the transfer of electrons to oxygen is not possible without sufficient oxygen. These electrons can be released under aerobic conditions during the experiment. The excess of electrons due to their previous accumulation under hypoxic conditions leads to a higher oxygen consumption than in healthy donors. Whole-body hyperthermia induces blood flow, which leads to the termination of hypoxia. The restored supply of oxygen stops the stress response of the cells, and autophagy returns to a ‘normal/healthy’ level. In addition, oxygen consumption increases even further due to the restored oxygen supply via the blood flow as well as the oxygen supply under aerobic experimental conditions.

Regardless of whether this hypothesis can be proven or not, our results definitively show an effect of whole-body hyperthermia on autophagy and the mitochondrial function in PBMCs from the nine participating ME/CFS patients. Altogether, we believe that heat treatment could be an effective way to treat not just one but multiple symptoms, allowing ME/CFS patients to have a better quality of life.

## 4. Materials and Methods

### 4.1. Study Design

**Figure 5:**
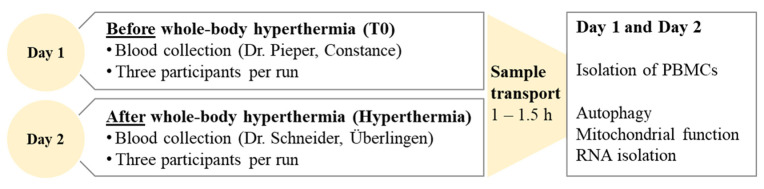
Study design. This study was conducted on two consecutive days. On the first day, blood was drawn to measure basal levels of autophagy, mitochondrial function and mRNA expression. Whole-body hyperthermia was performed on the second day. After the treatment, blood was taken again and transported to our laboratory to determine the same cellular mechanisms as on day 1.

Two consecutive days were required to investigate the influence of a session of whole-body hyperthermia on the central cellular mechanisms in ME/CFS patients. On the first day, the basal values of the patients were determined. For this purpose, blood was taken in the morning in Constance by Dr. Stefan Pieper and transported to our laboratory. peripheral blood mononuclear cells (PBMCs) were then isolated for the two cell tests to measure autophagy and mitochondrial function. In addition, RNA was isolated for subsequent qPCR analysis. On the second day, the patients were subjected to whole-body hyperthermia for one hour, with a maximal core body temperature (T_c_) of 39 °C. This treatment took place at the medical practice of Dr. Achim Schneider in Überlingen. After each session, blood was again taken from the participants and transported to our laboratory. Here, too, autophagy and mitochondrial function were measured directly after PBMC isolation, and RNA isolation was performed for the subsequent qPCR analysis. The entire procedure was performed three times with 3 participants each. The study design is shown in Figure 5.

### 4.2. Human Participants and Blood Collection

A total of nine ME/CFS patients (38 ± 10 years; f = 7, m = 2) participated in this study (Table 1). The ME/CFS patients were medically diagnosed using the diagnostic criteria of the Canadian consensus criteria [34] and the Fukuda criteria [35]. In addition, the severity of the disease was assessed using the Bell scale [36]. Hyperthermia treatment was performed upon medical recommendation, not for this study. The patients agreed to donate blood for research purposes before and after the therapy. To compare the measured values of ME/CFS patients with data from healthy volunteers (42 ± 11 years; f = 9, m = 6), data from other studies were used (Table 1) [17,37]. As with the ME/CFS patients, the control group contained more women than men and were in a similar age range. Blood collection was performed by a trained person using 9 mL blood collection tubes containing EDTA as anticoagulant (Sarstedt AG & Co. KG, Nümbrecht, Germany) and transported at room temperature. PBMC isolation was performed within 2 h of collection.

### 4.3. Whole-Body Hyperthermia Treatment

The heat therapy was carried out with the whole-body hyperthermia system ‘IRATHERM^®^1000M’ (Von Ardenne Institute of Applied Medical Research GmbH, Dresden, Germany). This system enables a rapid increase in core body temperature (to 39 °C in approx. 45 min) through uniform irradiance over the entire patient using 6 water-filtered infrared-A (wIRA) radiant heaters. Infrared-A radiation is short-wave IR radiation in the wavelength range from 780 to 1400 nm and is generated by a light bulb. One of the special features of this emitter is that it is encased in a layer of water. This layer of water serves as a filter, and the filtered radiation obtained is highly similar to Earth’s solar spectrum in the IR range [38]. The remaining IR-A has a high penetration depth into the human tissue, while at the same time, there is no thermal overload of the outer skin by IR-B and IR-C radiation. Therefore, water-filtered radiation is a proven method for performing hyperthermic applications [39]. The treatment session lasted one hour with a maximum core body temperature of 39 °C. During the session, the temperature was monitored via three temperature sensors (rectal, axillary and dermal) as well as by ear pulse and oxygen saturation.

### 4.4. Isolation of Human PBMCs

Human PBMCs were isolated from 36 mL of human blood using Leucosep tubes (Greiner Bio-One GmbH, Frickenhausen, Germany) according to the manufacturer’s instructions. PBMCs were resuspended in phosphate-buffered saline (PBS), and cell counts were determined using a Luna-FL™ automated cell counter (Logos Biosystems, Inc., Dongan-gu Anyang-si, Gyeonggi-do, South Korea). Measurements of mitochondrial respiration and autophagy-related protein light chain 3 (LC3-II) and RNA isolation were performed to determine mRNA expression within the freshly isolated PBMCs.

### 4.5. Antibody-Based Quantification of LC3-II

For the analysis of LC3-II, PBMCs were seeded in triplicate at a density of 500,000 cells/well in 200 µL RPMI1640 (Gibco^TM^, Waltham, MA, USA) without phenol red in a 96-well V-bottom plate (Merck KGaA, Darmstadt, Germany). For each condition tested, a 40 µM chloroquine (CQ) control was added in triplicate to evaluate the functionality of the assay. After cell seeding, the cell plates were placed in an incubator at 37 °C and 5% CO_2_ for CQ incubation. The Guava^®^ Autophagy LC3 Antibody-Based Detection Kit (Luminex Corporation, Austin, TX, USA) was used to stain the autophagy marker LC3 via antibody labelling. For this purpose, the plates were centrifuged at 300× *g* for 5 min. After centrifugation, the supernatant was discarded, and the cells were washed via resuspension in 100 µL 1× assay buffer. After a further centrifugation step (5 min, 300× *g*), 100 µL ‘Autophagy reagent B’ was added to the cells and centrifuged immediately (5 min, 300× *g*) without resuspending. After discarding the supernatant, the cells were resuspended in a 100 µL antibody solution containing 95 µL 1× assay buffer and 5 µL 20x anti-LC3 FITC (clone 4E12). Incubation was carried out at room temperature for 30 min in the dark. After incubation, the plates were centrifuged (5 min, 300× *g*) and washed with 100 µL assay buffer. After a final centrifugation step (5 min, 300× *g*), the cells were resuspended in 100 µL 1× assay buffer. The plates remained in the dark for 10 min before the cells were analysed via flow cytometry using the BD FACSVerse™ Flow Cytometry System (Becton, Dickinson and Company, Franklin Lakes, NJ, USA). By using a selective permeabilisation solution and washing steps, cytosolic LC3 was distinguished from autophagic LC3 by extracting the soluble cytosolic proteins, while LC3 sequestered in the autophagosome was protected.

### 4.6. Analysis of Mitochondrial Function—Seahorse XF Cell Mito Stress Test

The mitochondrial parameters were measured using the Seahorse Bioscience XFe96 Extracellular Flux Analyzer (Agilent Technologies Inc., Santa Clara, CA, USA). In preparation for sensor calibration, 200 µL XF Calibrant (Agilent Technologies Inc., Santa Clara, CA, USA) was added to each well of the XF sensor cartridge (Agilent Technologies Inc., Santa Clara, CA, USA) and incubated overnight at 37 °C without CO_2_. The XF96 cell culture microplate (Agilent Technologies Inc., Santa Clara, CA, USA) was coated with 10 µL 50 µg/mL poly-D-lysine (PDL) solution (Merck KGaA, Darmstadt, Germany) per well. A minimum of three wells were measured for each condition. A total of 225,000 PBMCs per well were seeded in 50 µL XF medium (Agilent Technologies Inc., Santa Clara, CA, USA) and centrifuged for 1 min at 250× *g* without brakes. All wells containing cells or background wells were filled with XF medium to a total volume of 180 µL. For bright-field cell imaging, the cell plate was placed in the Cell Imager Cytation 1 (Biotek Instruments GmbH, Bad Friedrichshall, Germany) and kept at 37 °C for 60 min. In the meantime, the sensor cartridge was loaded with the four cellular stressors, oligomycin (20 µL), carbonyl cyanide-4 (trifluoromethoxy)phenylhydrazone (FCCP, 22 µL) and a combination of rotenone, antimycin A and the cell staining reagent Hoechst (25 µL) (all Merck KGaA, Darmstadt, Germany). After injection, the final concentration of oligomycin was 1.25 μM, that of FCCP was 1.0 μM, that of rotenone and antimycin was 1.67 μM each and that of Hoechst was 8.0 µM. To calibrate the sensors, the 96-well sensor cartridge was inserted into the XFe96 Extracellular Flux Analyzer. After calibration, the utility plate was replaced with the cell plate to start measuring mitochondrial respiration in the PBMCs. The addition of Hoechst with the last injection allowed subsequent cell counting using the cell imager. Based on this cell count, the OCR could be normalised to 1000 cells per well. The data were analysed using the XF software ‘Agilent Seahorse Analytics’ (Agilent Technologies Inc., Santa Clara, CA, USA, version 1.0.0-749). A more detailed description of this method can be found in the methodological paper ‘Ex vivo Assessment of Mitochondrial Function in Human Peripheral Blood Mononuclear Cells Using XF Analyzer’ by Alica Schöller-Mann [40].

### 4.7. Determination of mRNA Expression in Human PBMCs

Approximately 10^6^ PBMCs were used for the RNA isolation, which was performed using the High Pure RNA Isolation Kit (Roche Diagnostics GmbH, Mannheim, Germany). The LunaScript^®^ Super Mix Kit (New England Biolabs, Ipswich, MA, USA) was used for cDNA synthesis. Both kits were used as described in the manufacturer’s instructions. The LightCycler^®^ 480 (Roche Diagnostics GmbH, Mannheim, Germany), in combination with the GoTaq^®^ qPCR Master Mix (Promega GmbH, Walldorf, Germany), was used for the quantitative polymerase chain reaction (qPCR) of multiple genes. Each qPCR consisted of 1 μL (0.5 pmoL/μL) of each primer (biomers.net GmbH, Ulm, Germany), 10 μL GoTaq^®^ qPCR Master and 8 μL cDNA. The qPCR analyses were performed in triplicate for each sample. The qPCR protocol used included the following steps: 5 min pre-incubation (95 °C), followed by 95 °C for 10 s, 62 °C for 10 s and 72 °C for 10 s, with fluorescence measurements at the end of the elongation phase. Melting-curve analysis was performed after 45 cycles. Ribosomal protein lateral peduncle subunit P0 (RPLP0) and β-actin were used as housekeeping genes to normalise the target genes. All the measured genes are listed in Table 2. Calculation of n-fold mRNA expression was performed using the 2^−ΔΔCt^ method [41].

### 4.8. Data Analyses

GraphPad Prism 10.3.1 for Windows (GraphPad Software Inc, San Diego, CA, USA) was used for statistical analyses and the creation of graphs. The results are presented as individual values, as grouped values with the mean ± SEM and the points of the individual values, as box-and-whiskers plots with the minimum to maximum and a line at the median and the individual point values, and as scatter plots with the mean ± SEM. Individual values are only shown descriptively, as the amount of data was too small for statistical analysis. All grouped data were tested for normal distribution using the Shapiro–Wilk test. The parametric *t*-test was used for normally distributed data and the non-parametric Wilcoxon test for non-normally distributed data. Statistical significance was defined as * *p* ≤ 0.05, ** *p* ≤ 0.01, *** *p* ≤ 0.001 and **** *p* ≤ 0.0001, or not significant (ns) for *p* > 0.05.

## Figures and Tables

**Table 1:** Information about the participants.

	*ME/CFS Patients*				*Healthy Donors*			
	Autophagy—Mitochondrial—mRNA Expression				Autophagy		Mitochondrial	
*Participant*	Sex	Age	Bell Scale	Symbol	Sex	Age	Sex	Age
*01*	f	31	20	●	f	61	f	30
*02*	f *	53 *	40 *	■	m	57	f	39
*03*	m	41	30	▲	f	61	f	29
*04*	m	50	50	▼	f	39	m	37
*05*	f	35	30–40	♦	m	42	m	44
*06*	f	32	30–40	◑	m	41	f	24
*07*	f	47	20	◨	f	34	-	-
*08*	f	32	40–60	◮	m	45	-	-
*09*	f	25	20–30	⧩	f	53	-	-

* This participant also took part in the first study (P03*) [17].

**Table 2:** Primer sequences.

Gene	Forward Primer (5′–3′)	Reverse Primer (5′–3′)
*RPLP0*	ccc gag aag acc tcc ttt tt	aga agg ggg aga tgt tga gc
*β-Actin*	gga ctt cga gca aga gat gg	agc act gtg ttg gcg tac ag
*ULK1*	ggt cac acg cca cat aac ag	gcc cca caa ggt gag aat aa
*ATG7*	acc cag aag aag ctg aac ga	ggt ggg agc act cat gtc aa
*BECN1*	ggc tga gag act gga tca gg	ctg tcc act gtg cca gat gt
*MAP1LC3B*	ggt gag aag cag ctt cct gt	aga ttg gtg tgg aga cgc tg
*AMPK1α*	ttc aag tga ttc tcc cgc ct	gaa gct gag gtg gtg gat ca
*FOXO3*	gca agc aca gag ttg gat ga	cag gtc gtc cat gag gtt tt
*SIRT1*	gca gat tag tag gcg gct tg	tct ggc atg tcc cac tat ca
*SIRT3*	cat gag ctg cag tga ctg gt	gag ctt gcc gtt caa cta gg
*TFAM*	ggg aag gtc tgg agc aga g	acg ctg ggc aat tct tct aa
*NDUFS1*	agg cag ttc tgc act cca aa	tcc atc tgc tcc cag gag aa
*SOD2*	cac cag cac tag cag cat gt	ggt gac gtt cag gtt gtt ca
*HSPA5*	ggt gaa aga ccc ctg aca aa	gtc agg cga ttc tgg tca tt
*IL-10*	gag aac agc tgc acc cac tt	gca tca cct cct cca ggt aa

## Data Availability

The original contributions presented in this study are included in the article/Supplementary Materials. Further inquiries can be directed to the corresponding author.

## Supplementary Materials

The supporting information can be downloaded at https://www.mdpi.com/article/10.3390/ijms26115339/s1.

## Abbreviations

The following abbreviations are used in this manuscript:

AMPK1α	protein kinase AMP-activated catalytic subunit alpha 1
APT	Adaptive Pacing Therapy
ATG	autophagy-related
ATP	adenosine triphosphate
BECN1	beclin 1
FOXO3	forkhead box O3
HIF	hypoxia-inducible factor
HSPA5	heat-shock protein family A (Hsp70) member 5
IL-10	interleukin-10
IR	infrared
MAP1LC3B	microtubule-associated protein 1 light-chain 3 beta
ME/CFS	myalgic encephalomyelitis/chronic fatigue syndrome
mRNA	messenger ribonucleic acid
NDUFS1	NADH-ubiquinone oxidoreductase 75 kDa subunit S1
OCR	oxygen consumption rate
PBMCs	peripheral blood mononuclear cells
PBS	phosphate-buffered saline
PEM	post-exertional malaise
ROS	reactive oxygen species
RPLP0	ribosomal protein lateral stalk subunit P0
SIRT1	sirtuin 1
SIRT3	sirtuin 3
SOD2	superoxide dismutase 2
T_c_	core body temperature
TFAM	mitochondrial transcription factor A
ULK1	unc-51-like autophagy activating kinase 1
wIRA	water-filtered infrared-A
