# Supplementary material for: Heat vs. Fatigue: Hyperthermia as a Possible Treatment Option for Myalgic Encephalomyelitis/Chronic Fatigue Syndrome (ME/CFS)
Source: Int J Mol Sci. 2025 Jun 1;26(11):5339. doi: 10.3390/ijms26115339 (PMC12154023; doi:10.3390/ijms26115339)
Supplement: Supplementary file 1 [file ijms-26-05339-s001.zip › ijms-3569040-supplementary.pdf]

Supplementary Materials

Table S1: Raw data of Figure 1

	Technical replicas of the LC3-II measurements								
	ME/CFS Patients						Healthy Donors		
	T0			Hyperthermia			T0		
Participant	Replica 1	Replica 2	Replica 3	Replica 1	Replica 2	Replica 3	Replica 1	Replica 2	Replica 3
01	409	414	430	264	262	271	328	324	321
02	441	441	442	283	296	305	218	247	241
03	255	251	266	240	233	248	218	220	216
04	435	422	385	337	348	357	378	377	375
05	321	330	339	303	309	310	323	308	307
06	315	316	295	284	284	288	363	347	339
07	333	337	334	319	312	312	368	325	355
08	404	401	405	332	347	337	287		295
09	321	311	312	261	251	248		226	229

Table S2: Raw data of Figure 2

	Technical replicates of mitochondrial function parameters (pmol/min/1000 cells)							
	ME/CFS Patients							
	T0				Hyperthermia			
Participant	Basal Resp.	ATP Prod.	Maximal Resp.	Spare Resp.	Basal Resp.	ATP Prod.	Maximal Resp.	Spare Resp.
01	0.444	0.428	2.218	1.774	0.673	0.691	2.813	2.140
01	0.528	0.488	2.295	1.767	0.817	0.811	2.889	2.072
01	0.477	0.440	1.842	1.366	0.922	0.874	3.393	2.471
01	0.512	0.504	2.117	1.606	0.892	0.873	3.654	2.761
01	-	-	-	-	0.782	0.746	2.755	1.973
01	-	-	-	-	0.888	0.845	3.440	2.552
01	-	-	-	-	0.784	0.678	2.536	1.752
Mean	0.49	0.46	2.12	1.63	0.51	0.54	1.83	1.32
SEM	0.02	0.02	0.1	0.1	0.03	0.03	0.14	0.11
02	0.485	0.453	1.446	0.961	0.462	0.458	1.418	0.957
02	0.469	0.460	1.612	1.144	0.422	0.454	1.633	1.211
02	0.422	0.402	1.217	0.796	0.588	0.586	2.379	1.790
02	0.502	0.462	1.278	0.776	0.657	0.647	2.249	1.592
02	0.514	0.489	1.633	1.119	0.431	0.474	1.464	1.033
02	0.458	0.417	1.404	0.946	0.534	0.542	1.797	1.263
02	0.536	0.491	1.478	0.942	0.627	0.671	2.423	1.796
02	0.466	0.444	1.550	1.084	0.495	0.541	1.695	1.200
02	0.364	0.417	1.243	0.878	0.414	0.446	1.437	1.023
Mean	0.47	0.45	1.43	0.96	0.82	0.79	3.07	2.25
SEM	0.02	0.01	0.05	0.04	0.03	0.03	0.16	0.14
03	0.720	0.725	2.824	2.105	1.090	1.036	4.264	3.174
03	0.781	0.739	2.700	1.919	1.296	1.233	4.309	3.014
03	1.012	0.994	3.260	2.248	1.331	1.264	5.430	4.100
03	0.878	0.843	2.896	2.018	1.388	1.333	5.236	3.847

03	0.810	0.794	3.216	2.406	1.370	1.308	4.989	3.618
03	0.894	0.843	3.325	2.431	1.343	1.298	4.743	3.401
Mean	0.85	0.82	3.04	2.19	1.3	1.25	4.83	3.53
SEM	0.04	0.04	0.11	0.09	0.04	0.04	0.2	0.17
04	0.416	0.417	1.009	0.593	0.628	0.618	1.787	1.159
04	0.401	0.410	0.762	0.361	0.667	0.681	2.107	1.440
04	0.425	0.401	0.478	0.053	0.626	0.629	1.941	1.315
04	0.427	0.393	0.989	0.561	0.644	0.648	2.048	1.404
04	-	-	-	-	0.572	0.577	1.540	0.968
Mean	0.42	0.41	0.81	0.39	0.63	0.63	1.88	1.26
SEM	0.006	0.005	0.12	0.12	0.02	0.02	0.1	0.09
05	0.450	0.441	1.401	0.951	0.467	0.449	1.138	0.671
05	0.470	0.460	0.758	0.287	0.504	0.504	1.828	1.323
05	0.452	0.474	1.103	0.651	0.561	0.548	2.082	1.521
05	0.443	0.456	1.058	0.615	0.563	0.545	1.646	1.083
05	-	-	-	-	0.519	0.529	1.782	1.263
05	-	-	-	-	0.449	0.450	0.993	0.544
05	-	-	-	-	0.484	0.512	1.178	0.694
Mean	0.45	0.46	1.08	0.63	0.51	0.51	1.52	1.01
SEM	0.006	0.007	0.13	0.14	0.02	0.02	0.16	0.14
06	0.287	0.319	0.362	0.075	1.037	1.017	4.279	3.242
06	0.269	0.321	0.339	0.070	0.954	0.973	3.699	2.745
06	-	-	-	-	1.075	0.991	4.584	3.509
Mean	0.28	0.32	0.35	0.07	1.02	0.99	4.19	3.17
SEM	0.008	0.0009	0.01	0.0024	0.04	0.01	0.26	0.22
07	0.492	0.498	1.883	1.391	0.632	0.564	2.270	1.638
07	0.549	0.552	1.790	1.241	0.886	0.852	3.094	2.208
07	0.545	0.512	1.846	1.301	0.737	0.727	2.500	1.764
07	0.580	0.558	1.996	1.417	0.711	0.667	2.228	1.518
07	0.484	0.496	1.757	1.273	0.690	0.665	2.887	2.197
07	-	-	-	-	0.641	0.625	2.242	1.601
07	-	-	-	-	0.649	0.645	1.807	1.158
Mean	0.53	0.52	1.85	1.32	0.71	0.68	2.43	1.73
SEM	0.02	0.01	0.04	0.03	0.03	0.03	0.17	0.14
08	0.422	0.413	1.296	0.874	1.042	0.899	4.887	3.845
08	0.487	0.482	2.030	1.543	1.130	1.041	4.751	3.621
08	0.646	0.622	2.035	1.389	1.219	1.068	5.532	4.313
08	0.676	0.689	1.893	1.217	1.095	0.990	5.187	4.093
08	0.599	0.662	1.601	1.003	-	-	-	-
08	0.600	0.604	1.829	1.229	-	-	-	-
08	0.515	0.534	1.210	0.695	-	-	-	-
08	0.535	0.555	1.181	0.646	-	-	-	-
Mean	0.56	0.57	1.63	1.07	1.12	1	5.09	3.97
SEM	0.03	0.03	0.13	0.11	0.04	0.04	0.17	0.15
09	0.927	0.881	3.872	2.945	1.807	1.542	7.709	5.902
09	0.891	0.815	3.549	2.659	2.065	1.830	7.878	5.813
09	1.092	1.087	3.969	2.877	1.841	1.738	5.614	3.773
09	0.912	0.983	3.819	2.907	2.010	1.797	7.879	5.869
09	1.121	1.067	4.111	2.990	1.618	1.493	6.800	5.182
09	0.879	0.784	3.584	2.706	1.589	1.443	6.505	4.916
09	1.040	0.946	3.197	2.157	1.399	1.324	5.893	4.494
Mean	0.98	0.94	3.73	2.75	1.76	1.6	6.9	5.14
SEM	0.04	0.04	0.12	0.11	0.09	0.07	0.36	0.3

	Healthy Donors			
	T0			
Participant	Basal Resp.	ATP Prod.	Maximal Resp.	Spare Resp.
01	0.267	0.282	1.807	1.540
01	0.307	0.309	1.421	1.114
01	0.241	0.248	1.465	1.224
01	0.228	0.267	1.095	0.866
Mean	0.42	0.44	1.47	1.04
SEM	0.01	0.02	0.07	0.08
02	0.471	0.507	1.520	1.049
02	0.402	0.426	1.536	1.134
02	0.416	0.381	1.733	1.318
02	0.377	0.421	1.415	1.038
02	0.422	0.450	1.417	0.995
02	0.461	0.463	1.175	0.714
Mean	0.26	0.28	1.45	1.19
SEM	0.02	0.01	0.15	0.14
03	0.274	0.221	1.100	0.826
03	0.308	0.279	1.036	0.728
03	0.250	0.252	1.047	0.797
03	0.279	0.270	0.981	0.702
Mean	0.28	0.26	1.04	0.76
SEM	0.02	0.03	0.05	0.06
04	0.325	0.404	1.527	1.202
04	0.399	0.439	2.490	2.091
04	0.405	0.407	2.151	1.746
04	0.399	0.437	2.461	2.062
Mean	0.38	0.42	2.16	1.78
SEM	0.02	0.01	0.22	0.21
05	0.486	0.553	1.991	1.505
05	0.493	0.547	1.640	1.146
05	0.524	0.591	2.501	1.977
05	0.370	0.460	1.563	1.194
Mean	0.47	0.54	1.92	1.46
SEM	0.03	0.03	0.21	0.19
06	0.485	0.653	2.436	1.951
06	0.364	0.361	1.521	1.157
06	0.521	0.472	2.402	1.881
06	0.536	0.505	2.783	2.248
Mean	0.48	0.5	2.29	1.81
SEM	0.04	0.06	0.27	0.23

Table S3: Raw data of Figure 3

	Ct values for the determination of mRNA expression																	
	Housekeeping Genes												Genes of interest					
	T0						Hyperthermia						T0			Hyperthermia		
	RPLP0			*β-Actin*			*RPLP0*			*β-Actin*			*ULK1*					
Participant	1	2	3	1	2	3	1	2	3	1	2	3	1	2	3	1	2	3
01	19.81	19.94	19.86	17.22	17.32	17.32	18.94	18.87	18.98	16.7	16.82	16.82	23.55	23.57	23.61	22.92	22.85	22.89
02	19.52	18.96	18.98	16.73	16.66	16.62	21.12	21.15	21.12	18.3	18.3	18.37	22.99	22.92	22.9	24.76	24.77	24.78
03	20.62	20.78	20.81	18.12	18.29	18.22	20.37	20.46	20.47	17.39	17.32	17.51	24.31	24.36	24.36	24.13	24.18	24.14
04	20.86	20.92	21.1	17.11	17.23	17.23	20.17	20.16	20.19	16.79	16.84	16.96	24.6	24.58	24.39	23.89	23.97	23.98
05	19.78	19.8	19.8	16.69	16.71	16.71	19.78	19.78	19.77	16.78	16.73	16.78	23.8	23.83	23.84	24.19	24.19	24.19
06	20.4	20.4	20.6	18.19	18.25	18.25	20.02	20.08	20.01	18.67	18.7	18.63	24.49	24.57	24.58	23.77	23.74	23.78
07	20.92	20.99	21.07	18.06	17.99	17.89	19.85	19.97	20.1	17.54	17.56	17.23	24.51	24.64	24.62	24.72	24.72	24.69
08	20.06	20.18	20.29	17.73	17.84	17.77	19.63	19.84	19.7	17.33	17.16	17.26	24.23	24.17	24.32	24.09	24.09	23.99
09	20.2	20.26	20.51	17.24	17.16	17.38	20.87	20.95	21.07	18.31	18.16	18.21	24.09	24.14	24.26	24.85	24.78	24.86
	RPLP0			*β-Actin*			*RPLP0*			*β-Actin*			*BECN1*					
01	19.81	19.94	19.86	17.22	17.32	17.32	18.94	18.87	18.98	16.7	16.82	16.82	24.53	24.37	24.53	23.23	23.33	23.33
02	19.52	18.96	18.98	16.73	16.66	16.62	21.12	21.15	21.12	18.3	18.3	18.37	23.51	23.48	23.39	25.34	25.3	25.31
03	20.62	20.78	20.81	18.12	18.29	18.22	20.37	20.46	20.47	17.39	17.32	17.51	25.47	25.39	25.35	25.13	25.23	25.16
04	20.86	20.92	21.1	17.11	17.23	17.23	20.17	20.16	20.19	16.79	16.84	16.96	25.66	25.69	25.58	24.59	24.52	24.56
05	19.78	19.8	19.8	16.69	16.71	16.71	19.78	19.78	19.77	16.78	16.73	16.78	24.7	24.75	24.73	24.27	24.35	24.36
06	20.4	20.4	20.6	18.19	18.25	18.25	20.02	20.08	20.01	18.67	18.7	18.63	25.09	24.87	25.01	24.86	24.74	24.73
07	20.92	20.99	21.07	18.06	17.99	17.89	19.85	19.97	20.1	17.54	17.56	17.23	25.62	25.78	25.67	25.25	25.02	25.19
08	20.06	20.18	20.29	17.73	17.84	17.77	19.63	19.84	19.7	17.33	17.16	17.26	25.2	25.28	25.34	25.21	25.2	25.13
09	20.2	20.26	20.51	17.24	17.16	17.38	20.87	20.95	21.07	18.31	18.16	18.21	25.24	25.52	25.18	26.42	26.31	26.28
	RPLP0			*β-Actin*			*RPLP0*			*β-Actin*			*ATG7*					
01	19.81	19.94	19.86	17.22	17.32	17.32	18.94	18.87	18.98	16.7	16.82	16.82	25.49	25.8	25.97	25.49	25.31	25.49
02	19.52	18.96	18.98	16.73	16.66	16.62	21.12	21.15	21.12	18.3	18.3	18.37	24.07	24.2	24.16	25.32	25.31	25.3
03	20.62	20.78	20.81	18.12	18.29	18.22	20.37	20.46	20.47	17.39	17.32	17.51	25.88	25.99	26.09	25.17	25.35	25.4
04	20.86	20.92	21.1	17.11	17.23	17.23	20.17	20.16	20.19	16.79	16.84	16.96	25.6	25.4	25.66	24.86	25.68	25.61
05	19.78	19.8	19.8	16.69	16.71	16.71	19.78	19.78	19.77	16.78	16.73	16.78	24.92	24.89	24.94	25.07	25.04	25.16
06	20.4	20.4	20.6	18.19	18.25	18.25	20.02	20.08	20.01	18.67	18.7	18.63	25.16	25.25	25.11	25.59	25.63	25.62
07	20.92	20.99	21.07	18.06	17.99	17.89	19.85	19.97	20.1	17.54	17.56	17.23	26.37	26.58	26.38	25.33	25.21	25.23
08	20.06	20.18	20.29	17.73	17.84	17.77	19.63	19.84	19.7	17.33	17.16	17.26	25.35	25.64	25.62	25.33	25.49	25.4
09	20.2	20.26	20.51	17.24	17.16	17.38	20.87	20.95	21.07	18.31	18.16	18.21	25.24	25.35	25.31	25.98	25.93	25.77
	RPLP0			*β-Actin*			*RPLP0*			*β-Actin*			*MAP1LC3B*					
01	19.81	19.94	19.86	17.22	17.32	17.32	18.94	18.87	18.98	16.7	16.82	16.82	23.63	23.84	23.8	22.9	23.08	23.06
02	19.52	18.96	18.98	16.73	16.66	16.62	21.12	21.15	21.12	18.3	18.3	18.37	22.59	22.63	22.65	24.01	24.12	24.05
03	20.62	20.78	20.81	18.12	18.29	18.22	20.37	20.46	20.47	17.39	17.32	17.51	24.5	24.5	24.49	24.25	24.15	24.38
04	20.86	20.92	21.1	17.11	17.23	17.23	20.17	20.16	20.19	16.79	16.84	16.96	24.16	24.3	24.36	23.23	23.36	23.55

05	19.78	19.8	19.8	16.69	16.71	16.71	19.78	19.78	19.77	16.78	16.73	16.78	23.59	23.57	23.59	22.65	22.63	22.65
06	20.4	20.4	20.6	18.19	18.25	18.25	20.02	20.08	20.01	18.67	18.7	18.63	23.59	23.72	23.72	23.12	23.27	23.24
07	20.92	20.99	21.07	18.06	17.99	17.89	19.85	19.97	20.1	17.54	17.56	17.23	24.27	24.37	24.47	23.6	23.57	23.52
08	20.06	20.18	20.29	17.73	17.84	17.77	19.63	19.84	19.7	17.33	17.16	17.26	23.29	23.55	23.35	23.08	23.03	23.09
09	20.2	20.26	20.51	17.24	17.16	17.38	20.87	20.95	21.07	18.31	18.16	18.21	23.58	23.66	23.54	24.08	24.09	24.19
	RPLP0			*β-Actin*			*RPLP0*			*β-Actin*			*AMPK1α*					
01	19.81	19.94	19.86	17.22	17.32	17.32	18.94	18.87	18.98	16.7	16.82	16.82	23.35	23.36	23.5	22.2	22.15	22.09
02	19.52	18.96	18.98	16.73	16.66	16.62	21.12	21.15	21.12	18.3	18.3	18.37	22.16	22.13	22.16	23.88	23.87	23.89
03	20.62	20.78	20.81	18.12	18.29	18.22	20.37	20.46	20.47	17.39	17.32	17.51	24.31	24.29	24.41	24.32	24.23	24.19
04	20.86	20.92	21.1	17.11	17.23	17.23	20.17	20.16	20.19	16.79	16.84	16.96	24.73	24.5	24.6	23.86	23.77	23.74
05	19.78	19.8	19.8	16.69	16.71	16.71	19.78	19.78	19.77	16.78	16.73	16.78	23.78	23.64	23.84	23.75	23.7	23.76
06	20.4	20.4	20.6	18.19	18.25	18.25	20.02	20.08	20.01	18.67	18.7	18.63	23.68	23.84	23.85	23.54	23.4	23.26
07	20.92	20.99	21.07	18.06	17.99	17.89	19.85	19.97	20.1	17.54	17.56	17.23	24.29	24.21	24.29	23.09	23.06	22.89
08	20.06	20.18	20.29	17.73	17.84	17.77	19.63	19.84	19.7	17.33	17.16	17.26	23.22	23.34	23.39	23.06	23.09	23.02
09	20.2	20.26	20.51	17.24	17.16	17.38	20.87	20.95	21.07	18.31	18.16	18.21	23.03	23.17	23.19	23.88	23.6	23.73
	RPLP0			*β-Actin*			*RPLP0*			*β-Actin*			*SIRT1*					
01	19.81	19.94	19.86	17.22	17.32	17.32	18.94	18.87	18.98	16.7	16.82	16.82	23.69	23.49	23.69	22.78	22.79	22.64
02	19.52	18.96	18.98	16.73	16.66	16.62	21.12	21.15	21.12	18.3	18.3	18.37	22.61	22.56	22.61	24.24	24.22	24.14
03	20.62	20.78	20.81	18.12	18.29	18.22	20.37	20.46	20.47	17.39	17.32	17.51	24.21	24.15	24.25	24.46	24.15	24.22
04	20.86	20.92	21.1	17.11	17.23	17.23	20.17	20.16	20.19	16.79	16.84	16.96	24.84	24.71	24.56	23.01	22.93	22.9
05	19.78	19.8	19.8	16.69	16.71	16.71	19.78	19.78	19.77	16.78	16.73	16.78	23.73	23.73	23.67	22.63	22.65	22.53
06	20.4	20.4	20.6	18.19	18.25	18.25	20.02	20.08	20.01	18.67	18.7	18.63	23.18	23.17	23.24	22.38	22.36	22.27
07	20.92	20.99	21.07	18.06	17.99	17.89	19.85	19.97	20.1	17.54	17.56	17.23	23.47	23.18	23.53	23.59	23.67	23.48
08	20.06	20.18	20.29	17.73	17.84	17.77	19.63	19.84	19.7	17.33	17.16	17.26	23.56	23.51	23.58	23	23.01	22.96
09	20.2	20.26	20.51	17.24	17.16	17.38	20.87	20.95	21.07	18.31	18.16	18.21	23.15	22.96	23.28	23.85	24.15	23.89
	RPLP0			*β-Actin*			*RPLP0*			*β-Actin*			*FOXO3*					
01	19.81	19.94	19.86	17.22	17.32	17.32	18.94	18.87	18.98	16.7	16.82	16.82	23.88	23.87	23.79	22.41	22.37	22.24
02	19.52	18.96	18.98	16.73	16.66	16.62	21.12	21.15	21.12	18.3	18.3	18.37	22.28	22.21	22.18	23.69	23.73	23.64
03	20.62	20.78	20.81	18.12	18.29	18.22	20.37	20.46	20.47	17.39	17.32	17.51	24.02	23.81	23.97	23.55	23.57	23.15
04	20.86	20.92	21.1	17.11	17.23	17.23	20.17	20.16	20.19	16.79	16.84	16.96	23.8	23.83	23.64	24.22	24.16	23.86
05	19.78	19.8	19.8	16.69	16.71	16.71	19.78	19.78	19.77	16.78	16.73	16.78	23.5	23.68	23.51	23.66	23.66	23.62
06	20.4	20.4	20.6	18.19	18.25	18.25	20.02	20.08	20.01	18.67	18.7	18.63	24.09	24.04	24.05	24.04	24.02	24
07	20.92	20.99	21.07	18.06	17.99	17.89	19.85	19.97	20.1	17.54	17.56	17.23	24.5	24.67	24.59	23.68	23.73	23.52
08	20.06	20.18	20.29	17.73	17.84	17.77	19.63	19.84	19.7	17.33	17.16	17.26	23.51	23.57	23.53	23.6	23.66	23.33
09	20.2	20.26	20.51	17.24	17.16	17.38	20.87	20.95	21.07	18.31	18.16	18.21	23.2	23.17	23.26	24.21	24.16	24.05
	RPLP0			*β-Actin*			*RPLP0*			*β-Actin*			*SOD2*					
01	19.81	19.94	19.86	17.22	17.32	17.32	18.94	18.87	18.98	16.7	16.82	16.82	22.54	22.49	22.57	22.14	22.11	21.98
02	19.52	18.96	18.98	16.73	16.66	16.62	21.12	21.15	21.12	18.3	18.3	18.37	21.31	21.3	21.3	22.08	22.08	22.04
03	20.62	20.78	20.81	18.12	18.29	18.22	20.37	20.46	20.47	17.39	17.32	17.51	22.52	22.47	22.61	22.6	22.55	22.61
04	20.86	20.92	21.1	17.11	17.23	17.23	20.17	20.16	20.19	16.79	16.84	16.96	22.04	22.09	22.09	21.5	21.49	21.39

05	19.78	19.8	19.8	16.69	16.71	16.71	19.78	19.78	19.77	16.78	16.73	16.78	22.08	22.03	22.13	20.87	20.83	20.79
06	20.4	20.4	20.6	18.19	18.25	18.25	20.02	20.08	20.01	18.67	18.7	18.63	21.68	21.73	21.9	21.16	21.32	21.11
07	20.92	20.99	21.07	18.06	17.99	17.89	19.85	19.97	20.1	17.54	17.56	17.23	21.06	20.93	21.07	21.3	21.22	21.22
08	20.06	20.18	20.29	17.73	17.84	17.77	19.63	19.84	19.7	17.33	17.16	17.26	21.91	21.95	21.92	21.51	21.36	21.23
09	20.2	20.26	20.51	17.24	17.16	17.38	20.87	20.95	21.07	18.31	18.16	18.21	21.89	22.07	21.92	22.67	22.8	22.77
	RPLP0			*β-Actin*			*RPLP0*			*β-Actin*			*SIRT3*					
01	20.19	20.34	20.35	17.81	17.76	17.86	19.55	19.57	19.54	17.42	17.7	17.65	26.35	26.39	26.5	25.79	25.63	25.8
02	19.68	19.7	19.7	17.72	17.75	17.71	21.47	21.59	21.52	19.14	19.15	19.25	26.17	25.96	26.26	27.67	27.8	27.54
03	20.64	20.79	20.78	18.37	18.36	18.52	20.3	20.29	20.32	17.63	17.64	17.57	26.86	26.81	26.97	26.6	26.56	26.58
04	21.61	21.67	21.68	18.76	18.69	18.68	20.04	20.07	20.03	17.7	17.67	17.69	27.94	27.9	27.82	26.88	26.78	26.78
05	20.54	20.58	20.61	17.9	17.96	18.03	20.54	20.62	20.67	17.95	18.03	18.05	26.2	26.31	26.37	26.68	26.62	26.67
06	20.77	20.51	20.49	17.72	17.74	17.72	19.74	19.77	19.98	17.64	17.74	17.73	26.19	26.18	26.15	25.94	25.94	26.02
07	21.18	21.37	21.49	17.88	17.88	18.04	20.38	20.21	20.13	17.53	17.41	17.19	27.26	27.11	27.28	26.72	26.84	26.66
08	21.03	21.21	21.12	18.35	18.35	18.37	20.71	20.51	20.53	17.8	17.88	17.8	26.58	26.48	26.51	26.37	26.54	26.08
09	19.89	19.83	20.02	16.8	16.74	16.87	20.41	20.38	20.52	17.54	17.44	17.43	25.64	25.59	25.63	26.24	26.21	26.24
	RPLP0			*β-Actin*			*RPLP0*			*β-Actin*			*TFAM*					
01	20.19	20.34	20.35	17.81	17.76	17.86	19.55	19.57	19.54	17.42	17.7	17.65	26.52	26.29	26.58	26.34	26.16	25.95
02	19.68	19.7	19.7	17.72	17.75	17.71	21.47	21.59	21.52	19.14	19.15	19.25	26.3	26.18	26.49	27.63	27.72	27.88
03	20.64	20.79	20.78	18.37	18.36	18.52	20.3	20.29	20.32	17.63	17.64	17.57	26.68	26.74	26.93	26.68	26.95	26.71
04	21.61	21.67	21.68	18.76	18.69	18.68	20.04	20.07	20.03	17.7	17.67	17.69	27.2	27.16	27.14	26.24	26.22	26.14
05	20.54	20.58	20.61	17.9	17.96	18.03	20.54	20.62	20.67	17.95	18.03	18.05	26.6	26.73	26.67	26.69	26.78	26.78
06	20.77	20.51	20.49	17.72	17.74	17.72	19.74	19.77	19.98	17.64	17.74	17.73	26.2	26.02	25.99	25.92	25.95	25.86
07	21.18	21.37	21.49	17.88	17.88	18.04	20.38	20.21	20.13	17.53	17.41	17.19	27.09	27.16	27.57	26.22	25.54	26.29
08	21.03	21.21	21.12	18.35	18.35	18.37	20.71	20.51	20.53	17.8	17.88	17.8	26.87	26.65	26.68	26.57	26.44	26.17
09	19.89	19.83	20.02	16.8	16.74	16.87	20.41	20.38	20.52	17.54	17.44	17.43	24.97	25.15	25.17	25.05	25.16	25.18
	RPLP0			*β-Actin*			*RPLP0*			*β-Actin*			*NDUSF1*					
01	20.19	20.34	20.35	17.81	17.76	17.86	19.55	19.57	19.54	17.42	17.7	17.65	25.52	25.53	25.6	24.81	24.89	24.89
02	19.68	19.7	19.7	17.72	17.75	17.71	21.47	21.59	21.52	19.14	19.15	19.25	25.52	25.58	25.57	26.85	27.06	26.82
03	20.64	20.79	20.78	18.37	18.36	18.52	20.3	20.29	20.32	17.63	17.64	17.57	25.93	26.03	26.02	25.71	25.72	25.78
04	21.61	21.67	21.68	18.76	18.69	18.68	20.04	20.07	20.03	17.7	17.67	17.69	27.03	26.91	26.93	25.35	25.31	25.47
05	20.54	20.58	20.61	17.9	17.96	18.03	20.54	20.62	20.67	17.95	18.03	18.05	26.07	26.11	26.19	25.95	26.12	26.04
06	20.77	20.51	20.49	17.72	17.74	17.72	19.74	19.77	19.98	17.64	17.74	17.73	25.66	25.71	25.56	25.58	25.6	25.27
07	21.18	21.37	21.49	17.88	17.88	18.04	20.38	20.21	20.13	17.53	17.41	17.19	27.12	27.49	27.28	25.55	25.27	25.22
08	21.03	21.21	21.12	18.35	18.35	18.37	20.71	20.51	20.53	17.8	17.88	17.8	26.78	26.82	27	25.82	26.1	26.05
09	19.89	19.83	20.02	16.8	16.74	16.87	20.41	20.38	20.52	17.54	17.44	17.43	24.88	24.78	25.04	25.49	25.41	25.42
	RPLP0			*β-Actin*			*RPLP0*			*β-Actin*			*HSPA5*					
01	19.81	19.94	19.86	17.22	17.32	17.32	18.94	18.87	18.98	16.7	16.82	16.82	20.95	20.86	20.84	19.84	19.84	19.9
02	19.52	18.96	18.98	16.73	16.66	16.62	21.12	21.15	21.12	18.3	18.3	18.37	20.26	20.34	20.47	21.8	21.77	21.75
03	20.62	20.78	20.81	18.12	18.29	18.22	20.37	20.46	20.47	17.39	17.32	17.51	21.96	22.03	21.94	21.7	21.78	21.78
04	20.86	20.92	21.1	17.11	17.23	17.23	20.17	20.16	20.19	16.79	16.84	16.96	22.95	22.9	23.03	21.14	21.18	21.2

05	19.78	19.8	19.8	16.69	16.71	16.71	19.78	19.78	19.77	16.78	16.73	16.78	21.4	21.37	21.52	19.66	19.67	19.61
06	20.4	20.4	20.6	18.19	18.25	18.25	20.02	20.08	20.01	18.67	18.7	18.63	21.7	21.85	21.65	20.88	20.9	20.76
07	20.92	20.99	21.07	18.06	17.99	17.89	19.85	19.97	20.1	17.54	17.56	17.23	22.26	22.34	22.23	21.94	21.99	21.94
08	20.06	20.18	20.29	17.73	17.84	17.77	19.63	19.84	19.7	17.33	17.16	17.26	21.2	21.14	21.07	21.11	21.07	20.9
09	20.2	20.26	20.51	17.24	17.16	17.38	20.87	20.95	21.07	18.31	18.16	18.21	21.18	21.53	21.26	22.65	22.38	22.27
	RPLP0			*β-Actin*			*RPLP0*			*β-Actin*			*IL-10*					
01	19.81	19.94	19.86	17.22	17.32	17.32	18.94	18.87	18.98	16.7	16.82	16.82	27.84	27.82	27.9	26.51	26.49	27.09
02	19.52	18.96	18.98	16.73	16.66	16.62	21.12	21.15	21.12	18.3	18.3	18.37	27.2	27.16	27.26	28.53	28.63	28.71
03	20.62	20.78	20.81	18.12	18.29	18.22	20.37	20.46	20.47	17.39	17.32	17.51	28.65	28.68	28.78	28.76	28.8	28.5
04	20.86	20.92	21.1	17.11	17.23	17.23	20.17	20.16	20.19	16.79	16.84	16.96	29.47	29.58	29.55	28.75	28.78	28.89
05	19.78	19.8	19.8	16.69	16.71	16.71	19.78	19.78	19.77	16.78	16.73	16.78	27.66	27.68	27.64	26.01	26.05	26.08
06	20.4	20.4	20.6	18.19	18.25	18.25	20.02	20.08	20.01	18.67	18.7	18.63	29.28	29.06	29.03	28.73	28.86	28.61
07	20.92	20.99	21.07	18.06	17.99	17.89	19.85	19.97	20.1	17.54	17.56	17.23	28.92	28.69	28.99	29.05	28.76	28.61
08	20.06	20.18	20.29	17.73	17.84	17.77	19.63	19.84	19.7	17.33	17.16	17.26	27.83	27.92	27.91	26.89	27.02	26.9
09	20.2	20.26	20.51	17.24	17.16	17.38	20.87	20.95	21.07	18.31	18.16	18.21	28.81	29.09	28.76	29.06	29.15	29.2
